# A transiting giant planet in orbit around a 0.2-solar-mass host star

**DOI:** 10.1038/s41550-025-02552-4

**Published:** 2025-06-04

**Authors:** Edward M. Bryant, Andrés Jordán, Joel D. Hartman, Daniel Bayliss, Elyar Sedaghati, Khalid Barkaoui, Jamila Chouqar, Francisco J. Pozuelos, Daniel P. Thorngren, Mathilde Timmermans, Jose Manuel Almenara, Igor V. Chilingarian, Karen A. Collins, Tianjun Gan, Steve B. Howell, Norio Narita, Enric Palle, Benjamin V. Rackham, Amaury H. M. J. Triaud, Gaspar Á. Bakos, Rafael Brahm, Melissa J. Hobson, Vincent Van Eylen, Pedro J. Amado, Luc Arnold, Xavier Bonfils, Artem Burdanov, Charles Cadieux, Douglas A. Caldwell, Victor Casanova, David Charbonneau, Catherine A. Clark, Kevin I. Collins, Tansu Daylan, Georgina Dransfield, Brice-Olivier Demory, Elsa Ducrot, Gareb Fernández-Rodríguez, Izuru Fukuda, Akihiko Fukui, Michaël Gillon, Rebecca Gore, Matthew J. Hooton, Kai Ikuta, Emmanuel Jehin, Jon M. Jenkins, Alan M. Levine, Colin Littlefield, Felipe Murgas, Kendra Nguyen, Hannu Parviainen, Didier Queloz, S. Seager, Daniel Sebastian, Gregor Srdoc, R. Vanderspek, Joshua N. Winn, Julien de Wit, Sebastián Zúñiga-Fernández

**Affiliations:** 1 Department of Space and Climate Physics, Mullard Space Science Laboratory, University College London, Holmbury St Mary, UK; 2 Department of Physics, University of Warwick, Coventry, UK; 3 Facultad de Ingeniería y Ciencias, Universidad Adolfo Ibáñez, Peñalolén, Chile; 4 Millennium Institute of Astrophysics (MAS), Santiago, Chile; 5 El Sauce Observatory, Obstech, Coquimbo, Chile; 6 Department of Astrophysical Sciences, Princeton University, Princeton, NJ USA; 7 Centre for Exoplanets and Habitability, University of Warwick, Coventry, UK; 8 European Southern Observatory (ESO), Vitacura, Chile; 9 Astrobiology Research Unit, Université de Liège, Liège, Belgium; 10 Department of Earth, Atmospheric and Planetary Science, Massachusetts Institute of Technology, Cambridge, MA USA; 11 Instituto de Astrofísica de Canarias (IAC), La Laguna, Spain; 12 Oukaimeden Observatory, High Energy Physics and Astrophysics Laboratory, Cadi Ayyad University, Marrakech, Morocco; 13 Instituto de Astrofísica de Andalucía (IAA-CSIC), Glorieta de la Astronomía s/n, Granada, Spain; 14 Department of Physics & Astronomy, Johns Hopkins University, Baltimore, MD USA; 15 Univ. Grenoble Alpes, CNRS, IPAG, Grenoble, France; 16 Observatoire de Genève, Département d’Astronomie, Université de Genève, Versoix, Switzerland; 17 Center for Astrophysics ∣ Harvard & Smithsonian, Cambridge, MA USA; 18 Sternberg Astronomical Institute, M. V. Lomonosov Moscow State University, Moscow, Russia; 19 Department of Astronomy, Tsinghua University, Beijing, People’s Republic of China; 20 NASA Ames Research Center, Moffett Field, CA USA; 21 Komaba Institute for Science, The University of Tokyo, Tokyo, Japan; 22 Astrobiology Center, Mitaka, Japan; 23 Departamento de Astrofísica, Universidad de La Laguna (ULL), La Laguna, Spain; 24 Kavli Institute for Astrophysics and Space Research, Massachusetts Institute of Technology, Cambridge, MA USA; 25 School of Physics & Astronomy, University of Birmingham, Birmingham, UK; 26 Canada–France–Hawaii Telescope, Kamuela, HI USA; 27 Université de Montréal, Département de Physique, IREX, Montreal, Quebec Canada; 28 SETI Institute, Mountain View, CA USA; 29 NASA Exoplanet Science Institute, IPAC, California Institute of Technology, Pasadena, CA USA; 30 George Mason University, Fairfax, VA USA; 31 Department of Physics, Washington University, St Louis, MO USA; 32 McDonnell Center for the Space Sciences, Washington University, St Louis, MO USA; 33 Center for Space and Habitability, University of Bern, Bern, Switzerland; 34 LESIA, Observatoire de Paris, CNRS, Université Paris Diderot, Université Pierre et Marie Curie, Meudon, France; 35 AIM, CEA, CNRS, Université Paris-Saclay, Université de Paris, Gif-sur-Yvette, France; 36 Department of Multi-Disciplinary Sciences, Graduate School of Arts and Sciences, The University of Tokyo, Tokyo, Japan; 37 Bay Area Environmental Research Institute, Moffett Field, CA USA; 38 Cavendish Laboratory, Cambridge, UK; 39 Space Sciences, Technologies and Astrophysics Research (STAR) Institute, Université de Liège, Liège, Belgium; 40 Department of Physics and Kavli Institute for Astrophysics and Space Research, Massachusetts Institute of Technology, Cambridge, MA USA; 41 Department of Astronomy, Yale University, New Haven, CT USA; 42 Department of Physics, ETH Zurich, Zurich, Switzerland; 43 Department of Earth, Atmospheric and Planetary Sciences, Massachusetts Institute of Technology, Cambridge, MA USA; 44 Department of Aeronautics and Astronautics, MIT, Cambridge, MA USA; 45 Kotizarovci Observatory, Viskovo, Croatia

**Keywords:** Exoplanets, Exoplanets, Giant planets

## Abstract

Planet formation models indicate that the formation of giant planets is substantially harder around low-mass stars due to the scaling of protoplanetary disc masses with stellar mass. The discovery of giant planets orbiting such low-mass stars thus imposes strong constraints on giant planet formation processes. Here we report the discovery of a transiting giant planet orbiting a 0.207 ± 0.011 *M*_⊙_ star. The planet, TOI-6894 b, has a mass and radius of *M*_P_ = 0.168 ± 0.022 *M*_J_ (53.4 ± 7.1 *M*_⊕_) and *R*_P_ = 0.855 ± 0.022 *R*_J_ and probably includes 12 ± 2 *M*_⊕_ of metals. The discovery of TOI-6894 b highlights the need for a better understanding of giant planet formation mechanisms and the protoplanetary disc environments in which they occur. The extremely deep transits (17% depth) make TOI-6894 b one of the most accessible exoplanetary giants for atmospheric characterization observations, which will be key for fully interpreting the formation history of this notable system and for the study of atmospheric methane chemistry.

## Abstract (web summary)

Analysis of data from multiple instruments reveals a giant exoplanet in orbit around the 0.2-solar-mass star TOI-6894. The existence of this exoplanetary system challenges assumptions about planet formation and it is an excellent target for atmospheric characterization.

## Main

Core-accretion planet formation models predict that the ability to form a giant planet scales with the mass of the host star^1,2^. This is primarily because these models indicate that a large amount of solid material in protoplanetary discs is necessary for the formation of giant planets and that observations have demonstrated that the mass of solid material in a protoplanetary disc scales with the mass of the star^3,4^. Therefore, it is expected that stars less massive than the Sun will form fewer giant planets^2^. In fact, several studies have predicted that very low-mass stars (M_*_ ≤ 0.3 *M*_⊙_) will not be able to form giant planets^2,5–8^.

The discovery of exoplanets orbiting stars substantially less massive than the Sun (for example, ref. ^9^) and determining their frequency of occurrence (for example, ref. ^10^) are, therefore, critical tests of giant planet formation. Existing surveys have shown that giant planets must be very rare around mid-to-late M-dwarf stars (for example, refs. ^11,12^) but have not been able to provide robust occurrence rate measurements.

To test the predictions of the formation theories, we conducted a survey using photometric data from the Transiting Exoplanet Survey Satellite (TESS)^13^ to search for giant planets transiting low-mass host stars^14^. Among the planet candidates discovered by this survey was a candidate giant planet transiting the very low-mass star TOI-6894 (in ref. ^14^, the candidate was listed by its TIC designation, TIC-67512645).

## Results

### Observations

**Fig. 1:**
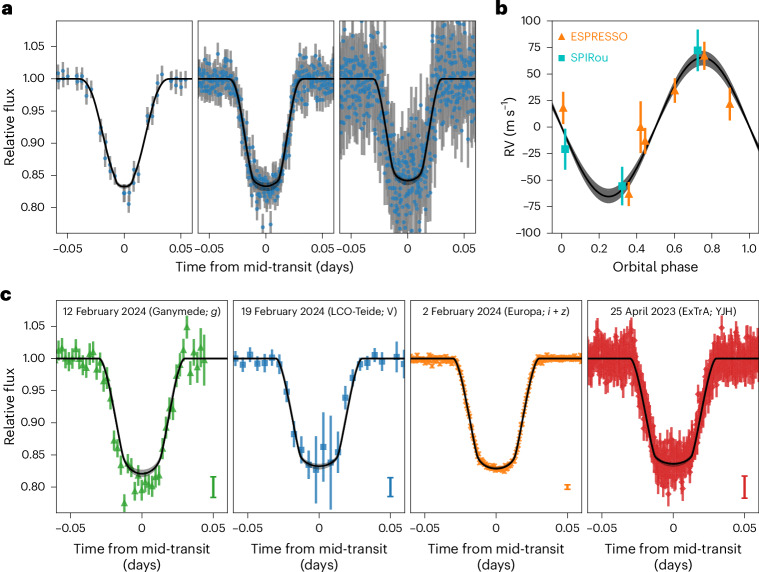
Transit light curves and RV data for TOI-6894 b. **a**, Phase-folded TESS photometric data at a cadence of 30 min (left), 10 min (middle) and 2 min (right) (blue points). **b**, Phase-folded RV data from ESPRESSO (orange triangles) and SPIRou (cyan squares). **c**, Selected ground-based follow-up photometric data. The panel annotations give the night on which the observations were taken, the facility that performed the observations (Europa and Ganymede are two SPECULOOS-South nodes) and the observing filter used. The best-fitting models obtained from the analysis in this work (Methods) are plotted as the black line in all panels. For all panels, the error bars are the reported uncertainties for each data point and the grey shaded regions give the 1*σ* uncertainty on the model. The error bars in the bottom right corners of the four panels in **c** denote the median error bar for the plotted observation. Note that all follow-up photometry, including the observations not plotted here, was included in the analysis. All follow-up photometry is plotted in Extended Data Fig. 1.

The 0.207 ± 0.011 *M*_⊙_ star TOI-6894 was initially observed by TESS from 18 February to 18 March 2020 in the full-frame images (FFIs) at a cadence of 30 min. A candidate transiting planet signal at a period of 3.37 days was reported by ref. ^15^ and was subsequently independently identified by ref. ^14^. Further shorter cadence monitoring by TESS, at a 10-min cadence from 6 November to 30 December 2021 and from 26 February to 26 March 2022 and at a 2-min cadence from 11 November to 7 December 2023, confirmed the presence of the transit signal and revealed it as a probable planet candidate (Fig. 1). Based on this extra monitoring and the results in ref. ^15^, the candidate was alerted as TOI-6894.01 by the TESS Science Office on 1 February 2024.

**Extended Data Fig. 1:**
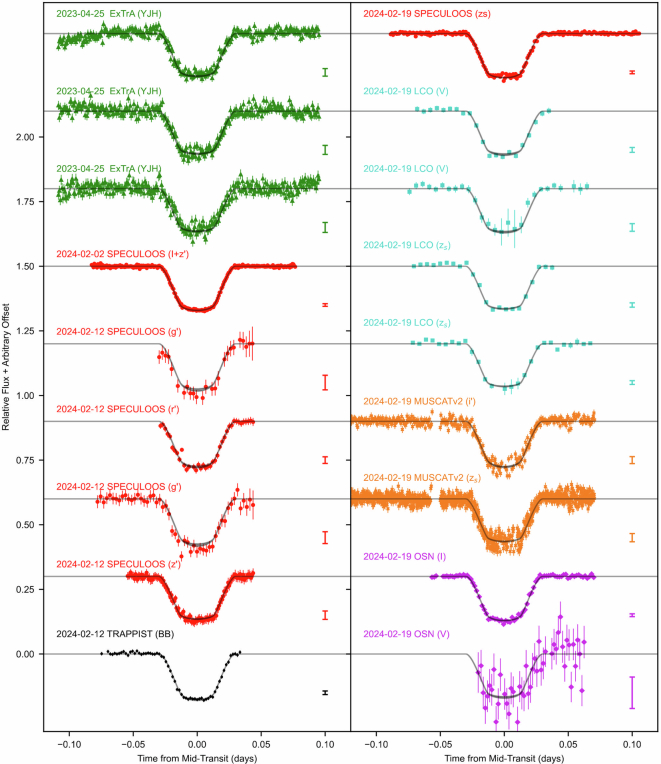
Ground-based light curve transit observations for TOI-6894 b. Each light curve is plotted individually and all light curves are offset from one another for clarity. The labels of each light curve give the date on which the observations were performed and the filter used for the observations. The different colours and markers denote the telescope used to obtain the observations: ExTrA (green triangles); SPECULOOS (red circles); TRAPPIST (black points); LCO (cyan squares); MUSCAT2 (orange crosses); OSN (purple diamonds). The the gray shaded regions provide the 1*σ* confidence region for the transit models. The errorbars provided are the reported uncertainties for all light curves except the MuSCAT2 data, for which the reported uncertainties were over-estimated and so we plot the rescaled uncertainties (see Methods) for clarity. For some observations the uncertainties are too small to be seen, and so we provide the median uncertainty for all observations as the errorbars plotted to the right of the corresponding observations.

**Extended Data Fig. 2:**
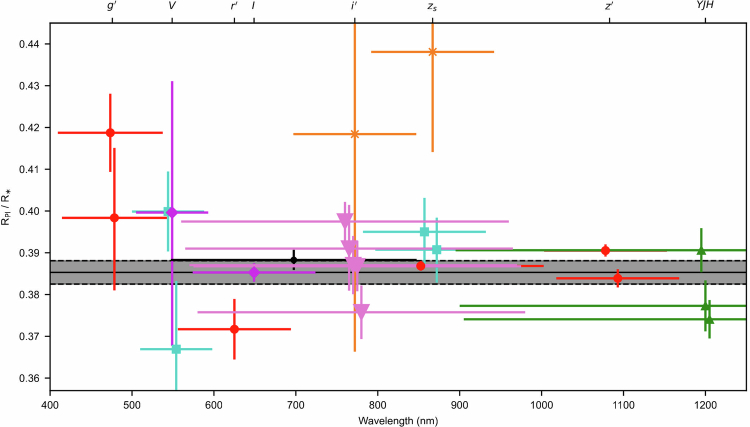
Individual RP/R* values obtained from transit fit analyses performed for each TESS sector and each individual ground-based transit light curve obtained. The x-axis plots the reference wavelength for each filter used. Where more than one result uses the same filter the points are offset slightly in the x-direction for clarity. The y-axis errorbars give the 1*σ* uncertainty from the transit analysis and the x-axis errorbars show the FWHM of the observing filter used. The upper axis shows the reference wavelengths for some of the filters used. The data point markers and colours are the same as for Extended Data Fig. 1, with the addition of the pink downward triangles for the TESS results. The solid black line and shaded grey region give the best fit RP/R* value and 1*σ* uncertainty from the global analysis.

**Extended Data Fig. 3:**
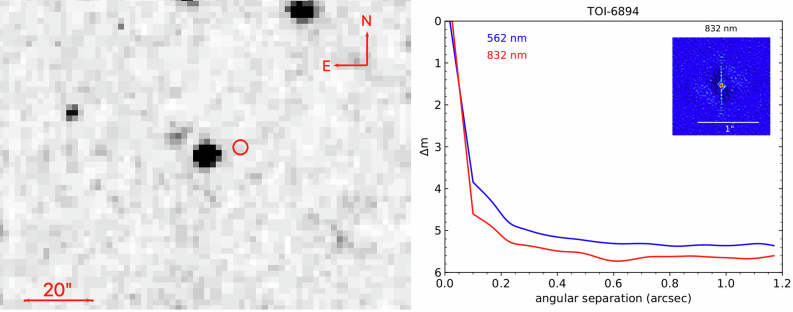
Observations to check for signs of blended companions. **Left**: The R-band 1-hour exposure time DSS image from the 48-Inch Palomar telescope observed in 1952 January 31. TOI-6894 is the star at the center of the image. The current sky location from Gaia DR3 is shown with a red circle. No background source is detected to the limit of the DSS plate (G = 19.5). **Right**: Contrast curves obtained using the ‘Alopeke speckle imager at Gemini North. The inset shows the obtained speckle image.

**Extended Data Fig. 4:**
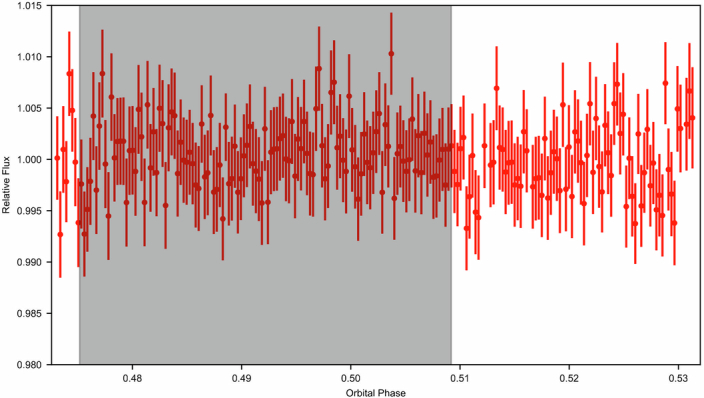
Checking for a secondary eclipse of TOI-6894 b. SPECULOOS observations taken during the prediction time of a secondary eclipse, with the error bars showing the reported photometric uncertainties. The gray shaded region gives the 1*σ* window for the estimated time of the occultation, accounting for the eccentricity posterior distribution. No significant secondary eclipse is observed.

Eclipsing binaries nearby to or in the background of the target star can blend into the photometric aperture and mimic a transiting exoplanet signal. The large pixel scale of the TESS cameras means that there is a higher likelihood of this occurring compared with other transit surveys. To investigate these scenarios, further transit observations of TOI-6894 b were obtained with several ground-based telescopes (Methods and Extended Data Fig. 1). These observations revealed that the transit signal is associated with the location of TOI-6894, thereby ruling out nearby eclipsing binary scenarios. We analysed each transit individually and found that the depth of the transit does not significantly vary with the wavelength, thereby ruling out background eclipsing binary scenarios that lead to chromatic transits (Methods and Extended Data Fig. 2). These ground-based observations also improved the determination of the planet radius and orbital ephemeris and are included in the full analysis of the system. Additionally, archival images dating back to 1952 show no background stellar contaminants at the current location of the system, and high-angular-resolution images show no associated sources in its immediate vicinity (Methods and Extended Data Fig. 3). Photometric observations taken during the secondary eclipse reveal no deep eclipse signal (Extended Data Fig. 4). All these together further validate the transit signal as genuine and probably due to a planetary companion.

**Extended Data Fig. 5:**
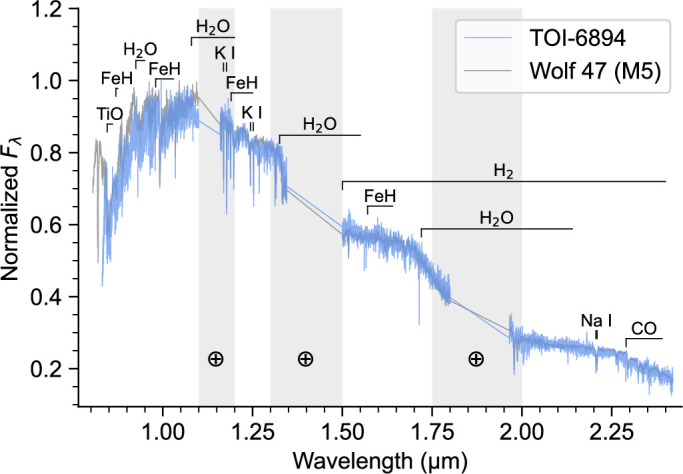
Magellan/FIRE spectrum of TOI-6894. The target spectrum (blue) is shown along with the spectrum of the best-fit M5 standard (grey). Regions of strong telluric absorption are shaded in grey, and prominent atomic and molecular features of M dwarfs are highlighted.

**Extended Data Fig. 6:**
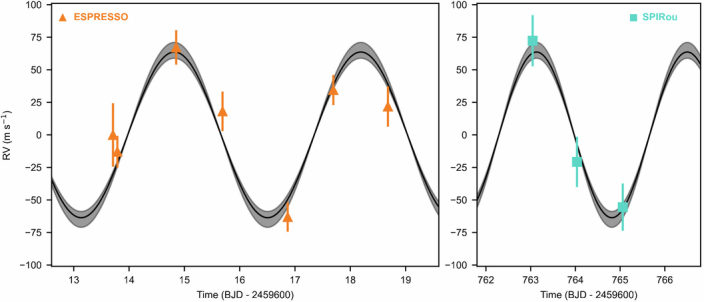
Radial velocity time series for TOI-6894. The ESPRESSO data is plotted in the left panel and the SPIRou data in the right, and the systemic radial velocity values (see Extended Data Table 2) have been subtracted from the respective radial velocity time series. The errorbars provided the 1*σ* radial velocity uncertainties yielded by the reduction pipelines. The symbols, colours, and model lines are the same as presented in Fig. 1b.

We collected a mid-resolution near-infrared spectrum of the host star using the folded-port infrared echellette (FIRE) spectrometer^16^ mounted on the Magellan telescope to assist with the stellar characterization and provide a measure of the stellar metallicity (Methods and Extended Data Fig. 5). High-resolution spectroscopic observations obtained using the ESPRESSO spectrograph at the Very Large Telescope^17^ revealed the variation of the stellar radial velocity (RV) at an orbital period and phase consistent with the photometric transit signal (Fig. 1 and Extended Data Fig. 6). Further spectroscopic observations with the SPIRou (Spectropolarimètre Infrarouge) spectrograph on the Canada–France–Hawaii Telescope (CFHT)^18^ corroborated this signal. We measured an RV semi-amplitude of 65.5 ± 8.3 m s^−1^, which is consistent with a planetary nature for the transiting body. Combining this semi-amplitude with directly observable parameters from the transit light curves alone^19^, we determined the surface gravity of the transiting body to be *g*_P_ = 5.73 ± 0.71 m s^−2^, consistent with a planetary-mass object.

### Analysis

We performed a joint analysis of all available observational data—all the TESS and ground-based photometric data, the RV measurements from ESPRESSO and SPIRou, broadband photometric measurements of the host star TOI-6894 and astrometric measurements from Gaia^20^—to determine the stellar and planetary parameters. The combined ESPRESSO spectra and the FIRE spectrum were used to derive priors on the stellar atmospheric parameters. The data were analysed using a differential evolution Markov chain Monte Carlo method (see Methods for details). The data and best-fitting model are shown in Fig. 1. (The full ground-based photometry is shown in Extended Data Fig. 1). To quantitatively assess the likelihood of any blended eclipsing binary scenarios, we modelled the available data as a blend between a bright M-dwarf star and a fainter blended eclipsing binary system (Methods). All blended binary scenarios produced significantly worse fits to the data than the scenario of a single star with a transiting planet. As such, our analysis confidently confirms the nature of the TOI-6894 system as a single star with a transiting planet, and we can confidently and quantitatively rule out all blended eclipsing binary scenarios.

**Fig. 2:**
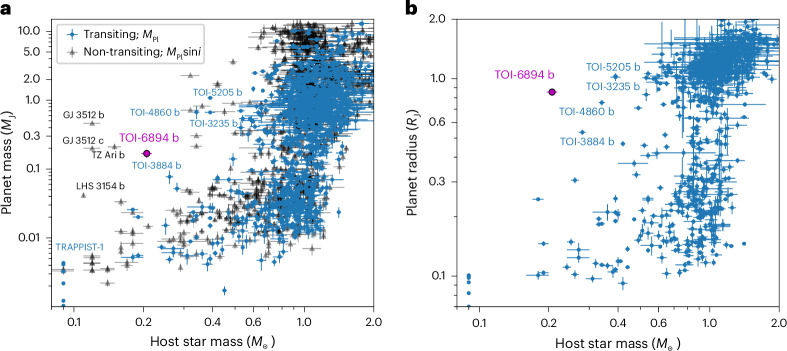
Placing TOI-6894 b in the context of known transiting planets. **a**, Masses (*M*_Pl_) or minimum masses (*M*_Pl_sin*i* where *i* denotes the orbital inclination) of the known population of planets discovered through the transit or RV method as a function of mass of the host star (data taken from the NASA Exoplanet Archive, accessed 16 May 2024). We plot transiting planets for which we have an absolute mass measurement as blue circles and non-transiting RV planets for which we have just a lower limit on the mass as the grey triangles. TOI-6894 b is plotted as the purple circle. The planets mentioned in the text and the transiting giant planets around mid M-dwarf stars are labelled for reference. The error bars are the 1*σ* uncertainty ranges on the plotted parameters. **b**, The same sample but showing the planet radii.

From our joint analysis, we found the host TOI-6894 to be a M5.0 ± 0.5 dwarf star with a radius of 0.2276 ± 0.0057 *R*_⊙_ and a mass of 0.207 ± 0.011 *M*_⊙_, a very low mass to host a giant planet, especially in the context of the known population of giant planets (Fig. 2). The low temperature of the host star (*T*_eff_ = 3,007 ± 58 K) results in TOI-6894 b having a relatively cool equilibrium temperature of just 417.9 ± 8.6 K, assuming an albedo *A* = 0.1 and efficient heat redistribution. TOI-6894 b has a mass of 0.168 ± 0.022 *M*_J_, which is just over half the mass of Saturn, and a radius of 0.855 ± 0.022 *R*_J_, which is just larger than Saturn. Here *M*_J_ is the mass of Jupiter and *R*_J_ its radius. Our analysis therefore reveals TOI-6894 b to be a low-density giant planet. TOI-6894 b orbits its host star with a period of 3.37077196 ± 0.00000059 days. The analysis yielded a measurement of the orbital eccentricity of 0.029 ± 0.030 with a 95% confidence upper limit of 0.094. The full set of derived parameters for both the planet and star are set out in Tables 1 and 2.

**Table 1:** Stellar properties of TOI-6894

Property	Value	Source
**Astrometric properties**		
Right ascension	11 h 33 min 52.5890 s	Gaia DR3
Declination	+12° 27′ 03.9373″	Gaia DR3
*μ*_RA_ (mas y^−1^)	−146.897 ± 0.056	Gaia DR3
*μ*_dec._ (mas y^−1^)	22.227 ± 0.053	Gaia DR3
Parallax (mas)	13.684 ± 0.053	Gaia DR3
**Photometric properties**		
TESS (mag)	14.9046 ± 0.0078	TIC8
Gaia G (mag)	16.2813 ± 0.0011	Gaia DR3
Gaia B_P_ (mag)	18.125 ± 0.018	Gaia DR3
Gaia R_P_ (mag)	14.9967 ± 0.0022	Gaia DR3
J (mag)	13.169 ± 0.023	2MASS
H (mag)	12.486 ± 0.022	2MASS
K (mag)	12.207 ± 0.021	2MASS
W1 (mag)	12.020 ± 0.023	WISE
W2 (mag)	11.842 ± 0.022	WISE
W3 (mag)	11.16 ± 0.15	WISE
**Derived properties**		
*T*_eff_ (K)	3,007 ± 58	This work (Methods)
[Fe/H]	0.142 ± 0.087	This work (Methods)
log *g*	5.039 ± 0.011	This work (Methods)
M_*_ (*M*_⊙_)	0.207 ± 0.011	This work (Methods)
R_*_ (*R*_⊙_)	0.2276 ± 0.0057	This work (Methods)
ρ_*_ (g cm^−3^)	24.73 ± 0.93	This work (Methods)
L_*_ (*L*_⊙_)	0.00375 ± 0.00033	This work (Methods)
Distance (pc)	72.96 ± 0.29	This work (Methods)

Note that other identifiers for the star are TIC-67512645 and *Gaia* DR3 3917278287286247808. Two Micron All-Sky Survey (2MASS)^120^; Gaia DR3 (ref. ^20^); TESS input catalogue v.8 (TIC8)^121^; Wide-field Infrared Survey Explorer (WISE)^122^.

**Table 2:** Planetary properties of TOI-6894 b

Name	Symbol	Unit	Value
Transit midpoint time	*T*_C_	BJD (TDB)	2,460,313.411670 ± 0.000042
Orbital period	*P*	Days	3.37077196 ± 0.00000059
Radius ratio	*R*_P_/R_*_		0.3860 ± 0.0029
Scaled semimajor axis	*a*/R_*_		24.59 ± 0.31
Impact parameter	*b*		$0.17{7}_{-0.040}^{+0.031}$
Orbital inclination	*i*	Degrees	$89.5{8}_{-0.07}^{+0.10}$
Transit duration	*T*_dur_	Hours	1.4220 ± 0.0062
RV semi-amplitude	*K*	m s^−1^	65.5 ± 8.3
Orbital eccentricity	*e*		0.029 ± 0.030 (≤0.094)
Planet radius	*R*_P_	*R*_J_	0.855 ± 0.022
Planet mass	*M*_P_	*M*_J_	0.168 ± 0.022
Planet bulk density	*ρ*_P_	g cm^−3^	0.334 ± 0.043
Planet-to-star mass ratio	*M*_P_/M_*_		(7.8 ± 1.1) × 10^−4^
Planet surface gravity	*g*_P_	m s^−1^	5.73 ± 0.71
Semimajor axis	*a*	au	0.02604 ± 0.00045
Planet irradiation flux	*S*	erg cm^−2^ s^−1^	(7.54 ± 0.60) × 10^6^
Planet equilibrium temperature^a^	*T*_eq_	K	417.9 ± 8.6
Transmission spectroscopy metric	TSM		356 ± 58

^a^Assuming albedo *A* = 0.1

Using a retrieval framework for warm giant planets^21^ (Methods), we modelled the interior structure of TOI-6894 b. We calculated a metal mass fraction (the fraction of the total planet mass that is not hydrogen or helium) *Z*_P_ = 0.23 ± 0.02. From the measured stellar metallicity of [Fe/H] = 0.142 ± 0.087, we calculated a stellar metal mass fraction of Z_*_ = 0.0189 ± 0.0037, finding the planet to be metal-enriched compared to its host star, with a metal mass fraction a factor of 12 higher. We determined the metal mass content of TOI-6894 b to be *M*_metal_ = 12 ± 2 *M*_⊕_.

## Discussion

TOI-6894 b joins an emerging population of giant planets with low-mass stars discovered through RV observations—LHS 3154 b (ref. ^22^), GJ 3512 b (ref. ^23^), GJ 3512 c (ref. ^24^) and TZ Ari b (ref. ^25^)—whose presence poses strong challenges to currently held formation theories. In particular, the core-accretion model, one of the current leading mechanisms for giant planet formation, struggles to form planets with masses greater than 30 *M*_⊕_ around low-mass stars^2,6–8^. The classic view of giant planet formation through core accretion necessitates the formation of a massive core, which then triggers a phase of runaway gas accretion^26^. The primary hurdles to the formation of these planets are the limited amount of solid material within the protoplanetary disc with which to form a massive-enough core, with lower-mass stars, in general, hosting lower-mass discs^3^, along with the longer Keplerian timescales around these stars, which inhibits the ability to form a massive-enough core before the dispersal of the gas disc^1^.

With a sub-Saturn mass, however, TOI-6894 b may not have been required to undergo a phase of runaway gas accretion. Recent studies have proposed that sub-Saturn-mass planets began their formation through a core-accretion process but did not undergo runaway gas accretion^27^. Instead, an intermediate phase of heavy-element accretion occurred, accompanied by a steady accretion of gas onto the forming protoplanet^27^. Such a mechanism may provide a plausible pathway for the formation of TOI-6894 b without necessitating rapid core formation or a runaway gas accretion phase.

Both the classic core-accretion and the sub-Saturn formation mechanisms would still require a suitable heavy-element mass budget to be present in the protoplanetary disc to provide the 12 ± 2 *M*_⊕_ metal mass content of TOI-6894 b. The efficiency of giant planet formation, that is, the fraction of the solid material in the disc available to be used to form the planet, has been estimated to be around 10% (ref. ^28^), following which the formation of TOI-6894 b would require a total of 120 *M*_⊕_ of solids to have been present in the disc. From a sample of 70 class II protoplanetary discs around stars within the mass range 0.15–0.25 *M*_⊙_, the most massive has a dust mass of 58.6 *M*_⊕_ and just a further four have a measured dust mass greater than the 12 *M*_⊕_ metal content of TOI-6894 b (ref. ^4^). As such, from this simple mass budget argument, it would initially seem that the formation of TOI-6894 b cannot be reconciled with the current sample of known protoplanetary discs.

However, there are a number of important caveats to this argument. First, these disc masses are calculated from the emission flux received from discs at millimetre wavelengths. Solid material in the disc in the form of centimetre-sized or larger pebbles would be undetectable through these observations, leading to an underestimate of the disc dust mass^29^. Similarly, observations of younger class 0 and I discs have also shown these discs to have dust masses an order of magnitude higher than class II discs^30^, and it has been theorized that large protoplanets may form during the class 0/I phase of the protoplanetary disc^31^. Furthermore, the current estimates of the formation efficiency are uncertain and depend on a number of poorly constrained characteristics of the protoplanetary disc^32^. Moreover, given the rarity of planets such as TOI-6894 (ref. ^14^) and given the small sample size of low-mass star discs studied, it is not unexpected that we have not yet discovered a massive-enough disc to easily explain the formation of TOI-6894 b. Therefore, it is plausible that TOI-6894 b could have formed through a core-accretion-like mechanism, either the classic picture or the sub-Saturn variation. Further information about these formation mechanisms and the nature of protoplanetary discs around these low-mass stars is required to fully reconcile this planet with the formation theory. TOI-6894 b will stand as a key benchmark planet for anchoring future theoretical studies in these areas.

An alternative pathway for the formation of massive planets is direct formation through condensation from a gravitationally unstable disc^33^. This mechanism has been shown to be capable of forming massive planets around low-mass stars, including the planet GJ 3512 b (ref. ^23^). However, simulations provide differing conclusions on the feasibility of forming a planet like TOI-6894 b. One set of simulations of planet formation around low-mass stars produced very massive planets with masses ≥2 *M*_J_ (ref. ^34^). Therefore, these simulations indicate that TOI-6894 b could not have formed through this mechanism. Conversely, a different suite of simulations demonstrated that this mechanism can form exoplanets with masses in the range 0.1–0.3 *M*_J_ around 0.2 *M*_⊙_ protostars^35^. These simulations may, therefore, indicate that this mechanism is a plausible formation pathway for TOI-6894 b. As the authors of the second study note, there were large differences in the initial conditions assumed in the two suites of simulations for the protoplanetary discs. Therefore, this mechanism remains a plausible formation pathway for TOI-6894 b, although gaining further information about the nature of protoplanetary discs will be required before we can fully interpret the formation of TOI-6894 b through this mechanism.

One potential hurdle in explaining the formation of TOI-6894 b through gravitational instability comes from recent planet synthesis simulations^36^, which did not form any planet with a core mass greater than 5 *M*_⊕_. This is significantly less than the 12 ± 2 *M*_⊕_ metal mass content of TOI-6894 b. However, note that these simulations did not consider the subsequent accretion of solids onto the formed fragments, and so these simulations underestimate the final metal mass content of the planets. There is also the possibility that a substantial fraction of the metal constituents of TOI-6894 b may be present in its atmosphere and may have been delivered through the capture of planetesimals by the protoplanet^37^. Such a dispersal of the metal content within TOI-6894 b would reconcile the nature of the planet with potential formation through gravitational instability. Atmospheric characterization through transmission spectroscopy may enable us to measure the atmospheric metallicity of TOI-6894 (ref. ^38^), thereby also providing a more robust measurement of the core mass, whose estimate from interior structure models based on mass and radius alone is degenerate with the atmospheric metallicity^38^. Atmospheric characterization could, therefore, provide a pathway for determining whether gravitational instability remains a plausible formation mechanism for TOI-6894 b.

**Fig. 3:**
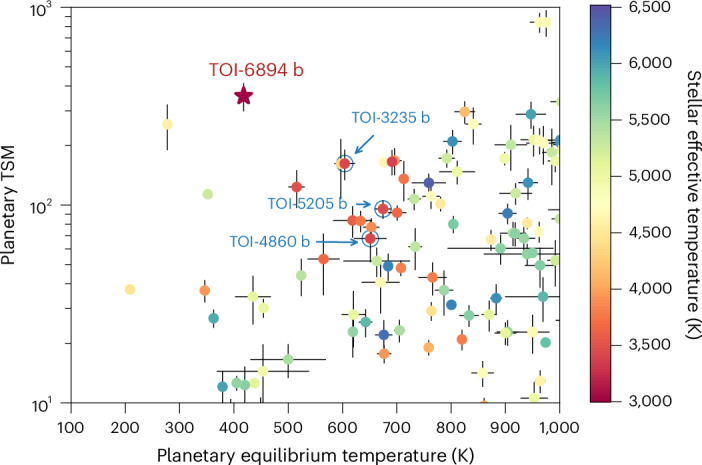
Atmospheric characterization potential of TOI-6894 b. TSM of known giant planets as a function of the planetary equilibrium temperature. The TSM (see ref. ^45^ for details) is an estimate of the expected S/N for transmission spectroscopy observations. The colour of the points denotes the effective temperature of the host star. The star symbol denotes TOI-6894 b. The values and error bars for the known population were calculated from the values provided in the NASA Exoplanet Archive. We highlight three known transiting giant planets with mid-M-dwarf host stars with the blue circles and arrows.

**Extended Data Fig. 7:**
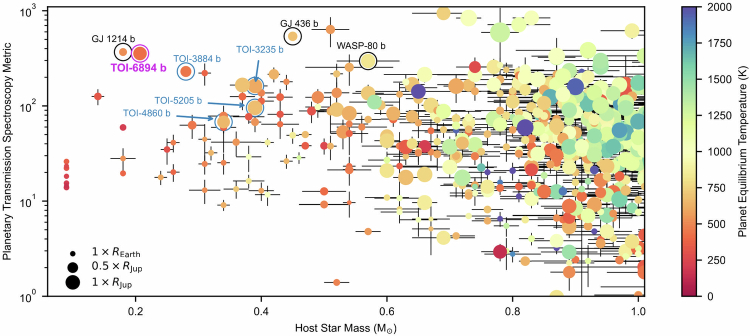
Transmission spectroscopy metric (TSM) as a function of host star mass for transiting exoplanets with mass measurements. Data and uncertainties are extracted from the NASA Exoplanet Archive, with the errorbars showing the reported 1*σ* uncertainties on each parameter. The points are coloured according to their planetary equilibrium temperature. The size of the points scale with the planetary radius. TOI-6894 b is highlighted by the magenta circle. We also highlight transiting giant and sub-giant planets with mid-M-dwarf host stars with the blue circles and arrows and other planets with high TSM already observed and/or scheduled on JWST with the black circles.

**Extended Data Fig. 8:**
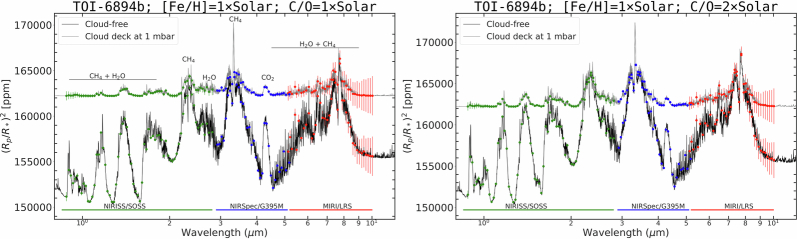
PandExo simulated transmission spectra of TOI-6894 b. **Left**: Clear and cloudy transmission spectra models assuming solar abundance are shown as solid lines. **Right**: Clear and cloudy models with the carbon-to-oxygen ratio enhanced by a factor of two. PandExo simulated observations with 1 transit for JWST NIRISS-SOSS, NIRSpec-G395M, and MIRI-LRS modes are also depicted, with their wavelength coverage indicated by coloured solid lines. The errorbars for both panels provide the estimated 1*σ* measurement uncertainties provided by PandExo.

TOI-6894 b is a key exoplanet for further exo-atmospheric investigations, beyond untangling the puzzling question of its formation. The equilibrium temperature of the planet makes it an intermediate object between the hot Jupiters that are being extensively observed by ground-based and space-based facilities^39,40^ and the cold gas giants of our own Solar System, namely Jupiter and Saturn. Based on its stellar irradiation, we expect that the planetary atmosphere is dominated by methane chemistry^41,42^. This alone would make TOI-6894 b a very valuable new discovery, as few descriptions of such examples have been published^43^, but what makes it truly special compared to previously studied objects such as WASP-80 b (refs. ^43,44^) is the combination of its particularly small host star, short orbital period and low planetary density for its cool equilibrium temperature. Combined, these make TOI-6894 b an extremely accessible giant planet with a low-mass host star for transmission spectroscopy observations (Fig. 3). The transmission spectroscopy metric (TSM)^45^ is a measure of the predicted signal-to-noise ratio (S/N) achieved for transmission spectroscopy observations. TOI-6894 b has a TSM of 356 ± 58, which is the highest of any giant planet with an equilibrium temperature *T*_eq_ ≤ 900 K or a host star mass M_*_ ≤ 0.7 *M*_⊙_ (Fig. 3, Extended Data Fig. 7 and Methods). Atmospheric models with and without clouds reveal that spectroscopic features in the transmission and emission spectra have expected amplitudes in excess of the primary transits of many planets (Methods and Extended Data Fig. 8). The detection of spectral features, the determination of the presence of clouds and the measurement of the atmospheric metallicity are possible even with medium-sized ground-based telescopes or from just a single transit observation with the James Webb Space Telescope (Methods). TOI-6894 b will, therefore, be a benchmark exoplanet in the study of methane-dominated atmospheres.

As a very low-mass star hosting a transiting giant planet, the TOI-6894 system is a benchmark system for our understanding of giant planet formation and for challenging the current theories, which struggle to explain its presence. The system is also highly amenable to transmission spectroscopy observations, through which we will be able to precisely determine both the atmospheric and interior composition of TOI-6894 b. The TOI-6894 system may, therefore, be a key exoplanetary system for determining the formation histories of giant planets, especially those with the lowest-mass host stars.

## Methods

### TESS observations

TOI-6894 (TIC-67512645) was observed by TESS^13^ during both the primary and extended missions. In the primary mission, TOI-6894 was observed in sector 22 (18 February to 18 March 2020), and in the extended mission, TOI-6894 was observed in sectors 45, 46 and 49 (6 November to 30 December 2021 and 26 February to 26 March 2022). Across all sectors, TOI-6894 was observed in the FFIs, and so TESS photometry is available at a cadence of 30 min for sector 22 and 10 min for the extended mission sectors. The TESS FFI photometry was processed by the TESS Science Processing Operation Center (SPOC)^46^. We accessed the data through the TESS-SPOC High-Level Science Product^47^. For our analysis, we used the PDCSAP light curves, which have been processed to remove spacecraft-related instrumental systematics^48–50^. The TESS light curves for TOI-6894 are displayed in Fig. 1. We display a cut-out pixel image of the area surrounding TOI-6894 in Supplementary Fig. 1.

### TESS candidate detection

TOI-6894 was included in a systematic transit search for giant planets with low-mass host stars in the FFI data from the TESS primary mission^14^. In short, this search detected periodic transit-like signals using the Astropy implementation of the box-fitting least squares algorithm^51,52^. It excluded clear false-positive scenarios and performed a transit-fitting analysis to identify probable giant planet candidates. Following these automated steps and some further manual vetting, TOI-6894 b was identified as a good quality giant planet candidate^14^. TESS-SPOC independently identified the signature of TOI-6894 b in transit searches of the FFI data from sectors 45, 46 and 49 using an adaptive matched filter^53–55^. After vetting the results of sector 49 with a modified version of TESS-ExoClass (https://github.com/christopherburke/TESS-ExoClass) for FFI targets^47^, TOI-6894 b was reported as a candidate^15^. The difference image centroid analysis^56^ for sector 46 constrained the location of the target star to be within 4.3 ± 2.5 arcsec of the transit source, substantially reducing the possibility of a nearby blended eclipsing binary scenario. TOI-6894 b was made a TESS object of interest on 1 February 2024.

### ExTrA observations

A full transit of TOI-6894 b was observed by ExTrA^57^, a low-resolution near-infrared (0.85–1.55 μm) multi-object spectrograph, on 25 April 2023. ExTrA was fed by three 60-cm-diameter telescopes at the European Southern Observatory’s (ESO’s) La Silla Observatory in Chile. Five fibres were positioned in the focal plane of each telescope to select light from the target and four comparison stars. Owing to the faintness of the target (*J* = 13.2 mag), we used the low-resolution mode of the spectrograph (*R* ≈ 20) and employed fibres with a 4" aperture to minimize the contribution of sky emission. The resulting ExTrA data were analysed using custom data-reduction software. The transit light curves from the three ExTrA telescopes are presented in Fig. 1 and Extended Data Fig. 1.

### SPECULOOS observations

Six full transits of TOI-6894 b were observed using various telescopes in the SPECULOOS^58–60^ 1m0-network at ESO Paranal Observatory in Chile and Teide Observatory in Tenerife^61^. All telescopes were equipped with a deep-depletion Andor iKon-L 2k × 2k CCD camera with a pixel scale of 0.35", resulting in a total field of view of 12" × 12". We collected the data during transits of TOI-6894 b on the nights of 2, 12 and 19 February 2024 in the *I* + *z*', Sloan-*g*', Sloan-*r'* and Sloan-*z*' filters and during an occultation of TOI-6894 b on the night of 7 February 2024 in the Sloan-*z*' filter. Science image processing and photometric extraction were performed using the PROSE pipeline^62^ (https://github.com/lgrcia/prose). The SPECULOOS data were detrended using external systematics variations related to time, the full-width at half-maximum of the point spread function, the sky background, the airmass and the *X* and *Y* pixel positions. The entire SPECULOOS transit photometry is plotted in Extended Data Fig. 1, and a selection is plotted in Fig. 1. The SPECULOOS occultation observation is plotted in Extended Data Fig. 4.

### TRAPPIST observations

A full transit of TOI-6894 b was observed with the TRAPPIST-South^63,64^ telescope on 12 February 2024 in the blue-blocking filter with an exposure time of 140 s. This is a 60-cm robotic Ritchey–Chretien telescope installed at ESO’s La Silla Observatory in Chile. It is equipped with a thermoelectrically cooled 2k × 2k FLI Proline CCD camera with a pixel scale of 0.65" and a field of view of 22' × 22' (refs. ^63,64^). Science image processing and photometric measurements were performed using the PROSE pipeline. The TRAPPIST photometry is plotted in Extended Data Fig. 1.

### Sierra Nevada Observatory observations

We observed TOI-6894 b on 19 February 2024 using the T150 at the Sierra Nevada Observatory (Observatorio de Sierra Nevada or OSN) in Granada, Spain. The T150 is a 150-cm Ritchey-Chrétien telescope equipped with a thermoelectrically cooled 2k × 2k Andor iKon-L BEX2DD CCD camera with a field of view of 7.9' × 7.9' and pixel scale of 0.232". We used the Johnson–Cousin *I* and *V* filters simultaneously with exposure times of 120 and 90 s, respectively. The photometric data were extracted using the AstroImageJ package^65^ and are plotted in Extended Data Fig. 1.

### LCOGT observations

TOI-6894 was also observed from the South African and Tenerife (Teide) nodes of the Las Cumbres Observatory Global Telescope network (LCOGT)^66^ using the 1-m telescopes on 19 February 2024. Both observations were carried out alternately in the *V* and *z*_s_ bands with exposure times of 300 and 70 s to cover the full transits. The observations were done with Sinistro cameras, which have a field of view of 26' × 26' and a pixel scale of 0.389". The raw images were automatically calibrated using the BANZAI pipeline^67^. We then performed the photometric analysis using the AstroImageJ software^65^ with an 8-pixel (3.1") or 5-pixel (1.9") aperture. The estimated point spread functions of the two observations are 1.85" and 1.65", respectively. All the LCO photometry is plotted in Extended Data Fig. 1, and the V-band photometry is also plotted in Fig. 1.

### MuSCAT2 observations

A full-transit observation of TOI-6894 b was collected on 19 February 2024 ut using MuSCAT2 (ref. ^68^) mounted on the 1.52-m Telescopio Carlos Sánchez at Teide Observatory, Tenerife, Spain. MuSCAT2 is a multicolour imager with a field of view of 7.4' × 7.4' and a pixel scale of 0.44". The observation was carried out simultaneously in four bands (*g*, *r*, *i* and *z*_s_). However, the *g*- and *r*-band data have a low S/N due to the large scatter induced by clouds. Therefore, we excluded these two datasets in our analysis. The *i*- and *z*_s_-band data were also impacted by the clouds but still had a sufficient S/N to be usefully included in the analysis. We carried out aperture photometry using the MuSCAT2 pipeline^69^ after dark-frame and flat-field calibration. The pipeline automatically finds the optimized aperture to minimize the photometric dispersion and then fits a transit model after accounting for instrumental systematic effects. The MuSCAT2 data are plotted in Extended Data Fig. 1.

### FIRE and Magellan

TOI-6894 was observed on the night of 26 February 2024 with the FIRE^16^ intermediate-resolution spectrograph operated at the 6.5-m Magellan Baade telescope, Las Campanas Observatory, Chile. We used a 0.6 × 7 arcsec slit that provided a spectral resolving power *R* = 4,500 in the wavelength range 0.82 < *λ* < 2.5 μm. We collected four 5-min-long exposures (a total integration of 20 min on source) with a ±1.5 arcsec nodding along the slit in the ABAB pattern under 0.65 arcsec full-width at half-maximum *J*-band atmospheric image quality. A telluric standard star 69 Leo (A0V) was observed right before the target and was used for flux calibration. We reduced the FIRE spectra of TOI-6894 using the FIRE bright source pipeline^70^, which outputs a flux-calibrated telluric-corrected spectrum merged from all 21 available echelle orders. A telluric correction algorithm^71^ fitted an observed stellar spectrum against a non-negative linear combination of synthetic stellar templates and a grid of Earth atmospheric transmission models computed using ESO SkyCalc^72^ for ESO La Silla, an observing site with very similar properties located geographically near to Las Campanas. The algorithm adjusted the final wavelength solution using telluric absorption lines to the final precision of about 0.3 km s^−1^. A spectrum is presented in Extended Data Fig. 5.

### ESPRESSO observations

We obtained spectroscopic observations of TOI-6894 using the ESPRESSO^17^ high-resolution, fibre-fed, cross-dispersed, echelle spectrograph to monitor the RV variations due to the orbit of its companion and to measure the mass of this transiting companion, thereby confirming its planetary nature. ESPRESSO is mounted at the Incoherent Combined Coudé Facility of ESO’s Very Large Telescope at Paranal Observatory in Chile. The observations were performed in high-resolution mode (*R* ≈ 140,000) as part of a programme dedicated to measuring the masses of giant planets around low-mass host stars (108.22B4.001; PI Jordan). We obtained seven spectra of TOI-6894 between 3 and 8 February 2022, using an exposure time of 2,400 s for each observation. The ESPRESSO data-reduction pipeline (v.2.3.5)^73,74^, as implemented within the EsoReflex environment^75^, was used to reduce the spectra. The RVs were measured using the dedicated Data Analysis Software (DAS; v1.3.6) for ESPRESSO. It measures the RVs by fitting a Gaussian model to the cross-correlation function. The cross-correlation function was derived by the DAS using an M4 stellar template, which most closely matches the spectral type of the host star. The ESPRESSO RVs are listed in Extended Data Table 2 and presented in Fig. 1 and Extended Data Fig. 6. In addition to this approach, we also measured the RVs using the SERVAL pipeline^76^, which applies the template-matching technique to obtain stellar RVs. The values obtained are consistent with the previous method within the uncertainties. We observed no significant correlation of the RV residuals with any of the activity indicators measured either by the DAS or SERVAL, which include the bisector span, Ca ii log *R*'_HK_, the differential linewidth and the chromatic index. We also computed the *H*_α_ index at both 0.6 and 1.6 Å using the ACTIN2 toolkit^77,78^, again finding no significance. We did, however, note relatively large variations in the absolute values of the bisector span, which are due to noise in the cross-correlation function and to the complex shape of those functions. We computed the periodogram of the ESPRESSO RVs. We found that there was a signal at the planetary orbital period, although we note that the significance of this signal from the RVs alone is low. So, although this is a strong detection of the RV signal due to TOI-6894 b given our previous knowledge of the planetary period from the TESS photometry, note that for a blind RV search, more RVs would be required to achieve a confident blind detection.

### SPIRou observations

We obtained three spectroscopic observations between 22 and 24 February 2024 (Programme 24AD02; PI Gan) for TOI-6894 using SPIRou^18^, which is installed on the 3.6-m CFHT. SPIRou is a fibre-fed, near-infrared, high-resolution spectropolarimeter (*R* ≈ 75,000) with a wavelength coverage between 0.98 and 2.5 μm. Because the host star is faint in the H-band, to avoid contamination, we chose to conduct the observations in dark mode without simultaneous drift calibration with the thermalized Fabry-Pérot etalon. All observations were collected with an exposure time of 1,800 s in an environment with airmass around 1.0 and seeing about 0.6", achieving S/N values of 89, 87 and 84 at order 44 (2.16 to 2.22 μm).

We reduced the data using APERO^79^ and extracted RV values through the line-by-line method from the telluric-corrected spectra^80^. The final RVs are the error-weighted average of all valid per-line velocities. The line-by-line method has been used in several recent TESS-related works to determine the mass of planets (for example, TOI-1759 b (ref. ^81^), TOI-2136 b (ref. ^82^), TOI-1452 b (ref. ^83^), TOI-1695 b (ref. ^84^) and TOI-4201 b (ref. ^85^)). The SPIRou RVs are plotted in Fig. 1 and Extended Data Fig. 6 and listed in Extended Data Table 2. Note that there is a systematic offset between the systemic velocity values obtained from the ESPRESSO and the SPIRou observations (Extended Data Table 2), which is due to the differences between the instrumental zero points and the wavelength coverage of the two instruments.

### Archival imaging

Because of the high proper motion of TOI-6894 (148.6 mas yr^−1^), archival imaging provides a useful check on line-of-sight blended neighbours. The 48-inch Oschin Telescope at Palomar Mountain, California, imaged TOI-6894 on the night of 31 January 1952 as part of the Palomar Observatory Sky Survey. The image was a 1-h exposure using the R-band filter. We accessed the digitized plate through the Space Telescope Science Institute’s Digitized Sky Survey (https://archive.stsci.edu/dss). The image shows TOI-6894 approximately 10" to the east of its current location (Extended Data Fig. 3), in agreement with the proper motion of the star as measured by Gaia (Table 1). An analysis of the Palomar image found no background source at the present position of TOI-6894 to the sensitivity of the photographic plate, which we estimated by cross-matching Gaia Data Release 3 (DR3) point sources to 5*σ* detections on the image. We could, therefore, rule out blended background sources to a magnitude limit of *G* = 19.5 mag.

### High-contrast imaging

Although blended background sources are ruled out by archival imaging, there is still the possibility of blending due to a co-moving companion. To investigate the possible multiplicity of TOI-6894, we obtained high-resolution imaging using the ’Alopeke speckle imager^86^ at Gemini North on 22 May 2024 (ut). We used 562/44 nm and 832/40 nm for the blue and red cameras, respectively. In each channel, we obtained 17,000 individual 60-ms frames, for a total integration time of 17 min in each band. Immediately thereafter, we observed a nearby star at a similar airmass to measure the speckle-transfer function. The data were reduced using the methods described by ref. ^87^. As shown in Extended Data Fig. 3, the ’Alopeke data rule out stellar companions within 1.2" and within ~5 mag at 562 nm and ~5.5 mag at 832 nm at most angular separations.

### Determining the stellar atmospheric parameters

The FIRE spectrum of TOI-6894 is shown in Extended Data Fig. 5. We used the SpeX Prism Library Analysis Toolkit^88^ to compare the spectrum to single-star spectral standards in the Infrared Telescope Facility Spectral Library^89,90^. We found the best match to the M5 standard Wolf 47, and thus, we adopted a spectral type of M5.0 ± 0.5 for TOI-6894. Following the approach of ref. ^91^, we used the relation between the equivalent widths of the K-band Na i and Ca i doublets and the H_2_O–K2 index^92^ to estimate the stellar metallicity^93^. This analysis yielded a super-solar iron abundance estimate of [Fe/H] = +0.240 ± 0.081.

An independent spectral analysis was performed on the ESPRESSO spectra using ODUSSEAS^94^, a machine-learning based code specifically designed for spectral analyses of M-dwarf stars (for example, ref. ^95^). From this analysis, we obtained values of [Fe/H] = −0.01 ± 0.10 and *T*_eff_ = 2,960 ± 66 K.

### Global analysis

A joint analysis was performed to derive and constrain the stellar and planetary parameters of the TOI-6894 system. For this analysis, we used all available data: the TESS transit discovery photometry and all the follow-up photometry, the ESPRESSO and SPIRou RV measurements, broadband photometry and astrometric data (for example, from ref. ^20^). The analysis followed the methods of refs. ^96–98^, and we direct the reader to those works for a more in-depth discussion. We present the key details of the analysis here. Mandel and Agol transit models^99^ were used to model the transit light curves. During the analysis, the limb-darkening coefficients were fitted as free parameters for each filter included, using Gaussian priors obtained from theoretical models^100–102^. A Keplerian orbit was assumed when modelling the RV measurements.

Broadband photometry from Gaia, 2MASS and WISE was included in the analysis to constrain the stellar parameters. We also used the parallax measurement from Gaia DR3 and stellar atmospheric parameters derived from the spectral analysis of the ESPRESSO and FIRE spectra. From these analyses, we adopted Gaussian priors of [Fe/H] = +0.240 ± 0.081 and *T*_eff_ = 2,960 ± 66 K for the joint analysis. We adopted the FIRE-derived [Fe/H] value, as the FIRE near-infrared spectrum provides a better S/N spectrum for determining the metallicity. However, note that we ran an independent analysis taking the ESPRESSO-derived metallicity as the prior range. The stellar and planetary parameters that this independent analysis yielded, including the derived stellar metallicity, are fully consistent with those reported in this paper. At each step of the analysis, the physical parameters of the host star were required to be consistent with the MIST stellar evolution models (v.1.2)^103–107^, allowing for systematic errors in these models following the methods of ref. ^98^.

A differential evolution Markov chain Monte Carlo procedure was used to fit the observations, using priors on the free parameters as listed in Supplementary Tables 1 and 2 (see also the discussion in ref. ^96^). After performing an initial fit to the data, we applied a sigma clipping to the light curves to remove outliers, and we rescaled the uncertainties to give *χ*^2^/degrees of freedom of 1 for each light curve. We then performed a second fit. For most of the light curves considered in this work, this did not make a statistically significant difference to the results of the fit, given the derived parameter uncertainties. The exception is the MuSCAT2 light curves, which had some large outliers, probably due to clouds impacting the observations. These outliers were removed before the final analysis. The planetary and stellar parameters reported in this work represent the median and 1*σ* uncertainty bounds calculated from the posterior distributions; these parameters are provided in Tables 1 and 2.

From our analysis, we found TOI-6894 b to be a transiting giant planet with a radius *R*_P_ = 0.855 ± 0.022 *R*_J_ (9.58 ± 0.25 *R*_⊕_) and a mass *M*_P_ = 0.168 ± 0.022 *M*_J_ (53.4 ± 7.1 *M*_⊕_). It orbits its host star with an orbital period *P* = 3.37077196 ± 0.00000059 days, semimajor axis *a* = 0.02604 ± 0.00045 au and an orbital eccentricity of 0.029 ± 0.030. The 95% upper limit placed on the orbital eccentricity is 0.094. Note that the eccentricity value we measured is very close to zero and consistent with zero within the errors. Therefore, we were unable to constrain the argument of the periastron of the orbit, *ω*. Because of the low eccentricity, we also repeated the analysis fixing the orbit to be circular. We compared the Bayesian information criterion of the two models, computed using only the RV data points as the light curve data do not contribute to constraining the eccentricity. We found a lower Bayesian information criterion for the eccentric model but with a difference of just 0.6, indicating that including the eccentricity as a free parameter is not strongly favoured by the data. However, note that the measured planet and stellar parameters are fully consistent between the two models, with the free-eccentricity model yielding slightly larger, more conservative uncertainties. As such, we chose to report the parameters from the free-eccentricity model in this paper.

We also found the host star TOI-6894 to be a very low-mass star with a mass and radius M_*_ = 0.207 ± 0.011 *M*_⊙_ and R_*_ = 0.2276 ± 0.0057 *R*_⊙_ and an effective temperature *T*_eff_ = 3,007 ± 58 K. This makes TOI-6894 one of the lowest-mass stars known to date to host a transiting giant planet, and just the fourth lowest-mass star to host any transiting planet. We compare the host star to other low-mass stars that host transiting planets in Supplementary Fig. 2.

### Blend analysis

To rule out the possibility that TOI-6894 is a blended stellar eclipsing binary system, we performed a blend analysis of the available observations following the method of ref. ^96^. To do so, we attempted to model the light curves, broadband catalogue photometry, spectroscopic atmospheric parameters and astrometric parallax of the object as a blend between a bright M-dwarf star and a fainter stellar eclipsing binary system. The parameters of the stars are constrained to follow the same MIST stellar evolution models used in the global joint analysis of the system. We found that a blended stellar eclipsing binary scenario is easily ruled out in favour of a single star with a transiting planet, with Δ*χ*^2^ = 1,600 between the best-fitting blended eclipsing binary model and the best-fitting transiting planet model. We also ruled out models consisting of an M-dwarf star with a transiting giant planet and a non-transiting, fainter M-dwarf companion with a mass down to the 0.1 *M*_⊙_ minimum stellar mass included in the MIST models. In this case, we found Δ*χ*^2^ = 140 between the best-fitting model with an unresolved stellar companion and the best-fitting model for a single star with a transiting planet. Note that all blended models considered have more free parameters than the single star plus planet model and are, thus, strongly disfavoured by any model selection criteria. Therefore, from this analysis we can confidently rule out any blend scenarios and can be confident that the TOI-6894 system is a single star with a transiting planet companion.

### Chromaticity analysis

We performed a further analysis to investigate whether the transit depth of TOI-6894 b varies with the wavelength of light in which the transit is observed. Such a chromatic variation would be evidence that the eclipse signals were due to a blended eclipsing binary or could point towards the presence of an unseen stellar companion. For this analysis, we performed a transit fit to each individual ground-based transit light curve obtained. We also performed a fit to each TESS sector individually. For these other transit analyses, we allowed only the planet-to-star radius ratio *R*_P_/R_*_, the quadratic limb-darkening coefficients and the out-of-transit flux baseline to vary. The remaining transit parameters—the time of the transit mid-point *T*_C_, *P*, *a*/R_*_ where *a* is the semi-major axis of the planet’s orbit and the planet’s orbital inclination *i*—were fixed to the best-fitting results from the global analysis. For the radius ratio, *R*_P_/R_*_, we used an uninformative uniform prior between 0 and 1. For the limb-darkening coefficients, we used a wide Gaussian prior with the best-fitting result from the global analysis as the mean and five times the uncertainty as the standard deviation. We plot the results from this analysis in Extended Data Fig. 2. We found no evidence from this analysis of a chromatic variation of the transit depth.

### Planet composition analysis

We modelled the interior structure of TOI-6894 b within a retrieval framework for warm giant planets^21^ that uses the forward models in ref. ^108^. We found from this analysis a metal mass fraction (the fraction of the planet mass that is not hydrogen or helium) *Z*_P_ = 0.23 ± 0.02. Note that the uncertainty quoted here is a statistical error based on the uncertainties on the stellar mass, radius and age. Combining with the overall mass of the planet, we found the metal mass content of TOI-6894 b to be *M*_metal_ = 12 ± 2 *M*_⊕_.

An empirical mass–radius relation for cool giant planets was derived in ref. ^109^ using the known population. From this known population, its mass–radius relation and the resulting dispersion in the population, the median planet radius for a 0.164 *M*_J_ planet is 0.67 *R*_J_ with a 1*σ* dispersion of ±0.18 *R*_J_. Therefore, although TOI-6894 b has a lower density than the median planet expected from the bulk population, its radius of *R*_P_ = 0.855 ± 0.022 *R*_J_ is consistent with the dispersion seen in the overall population to within a tolerance of 1*σ*.

### Search for more planets and detection limits

We analysed the 120 s of TESS data with the SHERLOCK package^110,111^. We refer the readers to refs. ^112,113^ for recent use of and searching strategies with this package. We first found a strong signal corresponding to the known 3.37-day planet, which enabled us to confirm independently of the SPOC pipeline the detectability of this planet in TESS data. We found no other signal hinting at extra transiting planets in orbital periods ranging from 0.5 to 15 days.

We then performed injection and retrieval experiments on this dataset to establish detection limits. We employed the MATRIX package^114^, which generated a sample of synthetic planets by combining a range of orbital periods, planetary radii and orbital phases injected into the data.

In particular, we generated 36,000 scenarios and searched them for transit-like features, mimicking the procedure conducted by SHERLOCK. From the results displayed in Supplementary Fig. 3, we conclude that the TESS data do easily allow us to detect large transiting planets (*R* > 5 *R*_⊕_) in short orbital periods (*P* < 6 days) with recovery rates of ~100%. Indeed, TOI-6894 b falls in this region. These planets become more challenging to detect for longer orbital periods, although doing so is still possible, with recovery rates between 40% and 80%. These results allowed us to conclude that the existence of such planets in the system is very unlikely. On the other hand, small transiting planets with sizes smaller than 4 *R*_⊕_ would be undetectable in the complete set of periods explored. Hence, we cannot offer any constraint on the existence of these planets in the system.

### Atmospheric characterization prospects

We expect TOI-6894 b to become a benchmark planet in the study of temperate H/He atmospheres. TOI-6894 b receives a stellar irradiation *S* = 5.50 ± 0.44 *S*_⊕_, which translates into an equilibrium temperature *T*_eq_ = 417.9 ± 8.6 K. This value assumes an albedo *A* = 0.1, like that of many hot and warm Jupiters^115^. At this temperature, it is widely expected the planet is dominated by methane chemistry, like WASP-80 b (refs. ^43,44^). Using these properties, we modelled possible atmospheres with and without clouds and with high and low C/O ratios, and we found that methane absorption features in the transmission spectrum of the planet would be expected to have amplitudes of 6,000, 9,000 and 11,000 ppm in the optical, near-infrared and mid-infrared, well in excess of any other giant to date, particularly for planets with a similarly low equilibrium temperature. This is mainly caused by two effects. The host star, TOI-6894, is small and transmission features are amplified by R_*_^−2^ and by the surprising low surface gravity of TOI-6894 b. To address the detectability of individual molecules within the TOI-6894 b planet spectrum, we used the methodology applied in refs. ^116–118^ for the transit geometry. We ran the James Webb Space Telescope PandExo noise model across a grid in number of transits from 1 to 100, which is sufficient to establish a simple S/N scaling relation, and we determined the S/N on the difference between the model spectrum and the fiducial spectrum. Our PandExo simulations of observations using the NIRISS/SOSS, NIRSpec/G395M and MIRI/LRS modes on TOI-6894 b showed that a single transit could suffice to retrieve abundances of key atmospheric species like methane, water and carbon dioxide, with a total expected S/N ≥ 100. We plot example transmission spectra obtained from PandExo in Extended Data Fig. 8. Furthermore, as illustrated in Extended Data Fig. 8, molecular absorption features should be detectable at wavelengths beyond 2 μm, even with a cloud deck at 1 mbar.

To place this planet in context, we calculated its TSM (see ref. ^45^ for details). The TSM can be used as a measure of how amenable a planet is to atmospheric characterization through transmission spectroscopy. We found that TOI-6894 b has a TSM of 356 ± 58. Comparing the TSM of TOI-6894 b to values for other known planets (Fig. 3 and Extended Data Fig. 7), we found TOI-6894 b to have the highest TSM of any giant planet with a host star less massive than 0.7 *M*_⊙_ and the second highest for any planet with a low-mass host star (M_*_ ≤ 0.4 *M*_⊙_), second only to GJ 1214 b. TOI-6894 b particularly stands out when considering other planets with a low equilibrium temperature.

Assuming that the planet has an albedo *A* = 0.1, we would expect its emission spectrum to be highly amenable to the detection of atmospheric features. As for transmission, we modelled possible emission spectra and found typical eclipse depths of 1,000–6,000 ppm in the mid-infrared. Studying the atmosphere of TOI-6894 b could provide easy access to an H/He atmosphere intermediate between those of hot Jupiters and the Jupiter in our Solar System. Studying its chemistry may help to refine atmospheric models. In addition, studying the atmosphere of a planet can provide further clues related to its formation history. The star is metal-rich ([Fe/H] = 0.142 ± 0.087), and it will be of interest to measure whether its atmosphere is too. Such measurements would reveal the true metal content of TOI-6894 b, thereby also revealing the composition of TOI-6894 b and giving clues about its formation history^38^.

### Reporting summary

Further information on research design is available in the Nature Portfolio Reporting Summary linked to this article.

## Supplementary information

Supplementary Information Supplementary Figs. 1–3 and Tables 1 and 2.

Reporting Summary

## Figures and Tables

**Extended Data Table 1:**
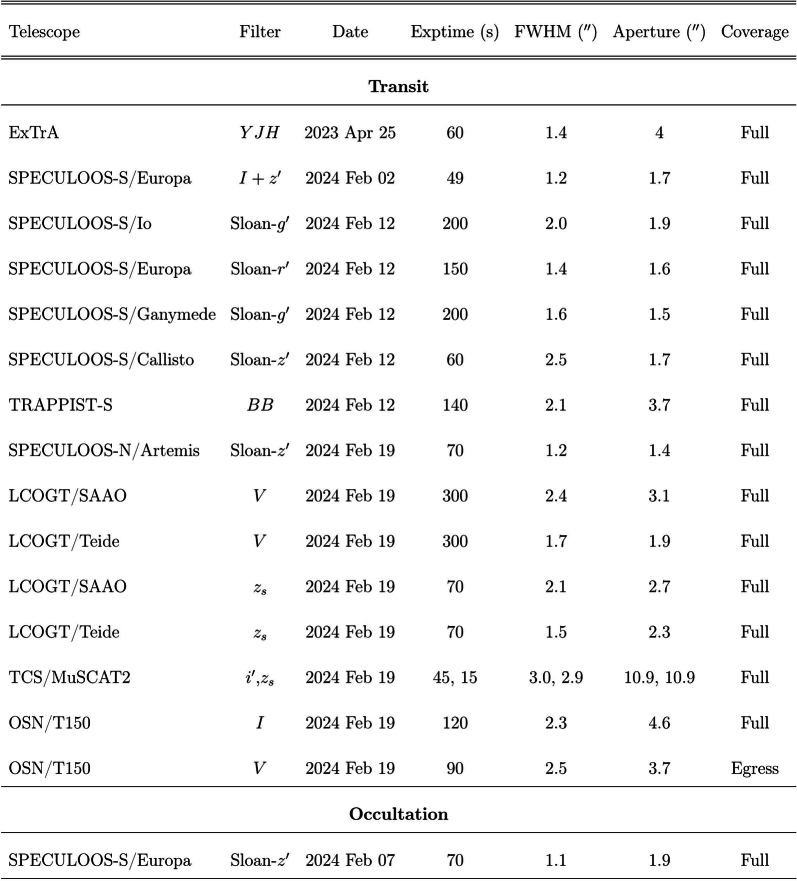
Ground-based follow-up observations log for TOI-6894 b

.

**Extended Data Table 2:**
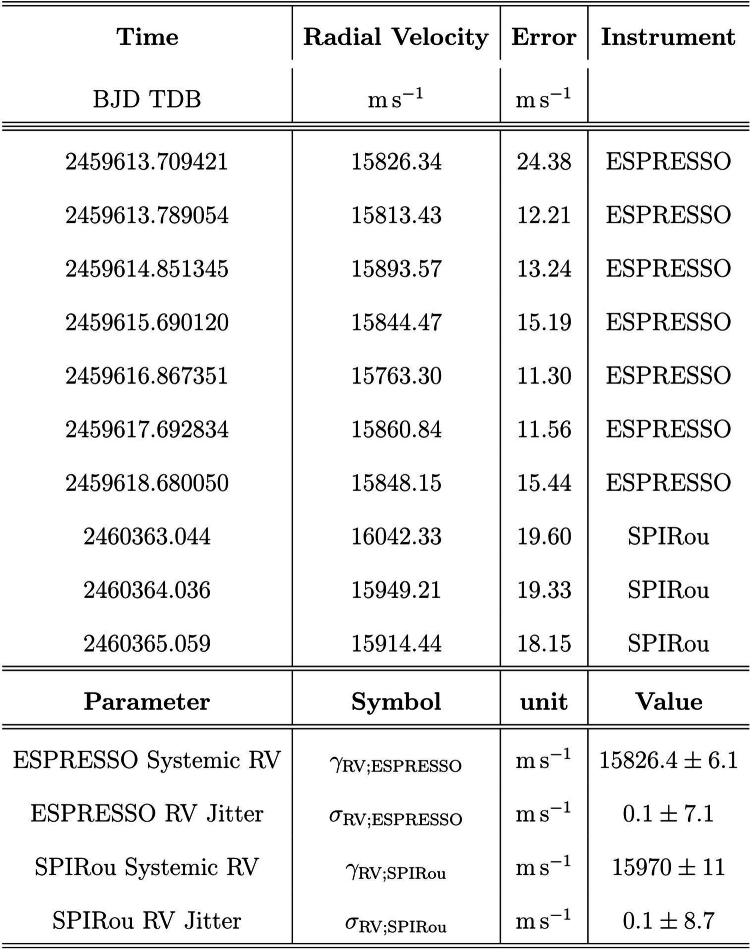
Radial velocity information for TOI-6894

We provide both the measured radial velocities and their uncertainties, as well as the systemic RV and jitter values determined for each instrument.

## Data Availability

The TESS-SPOC FFI photometry we used is publicly available as a high-level science product from the Mikulski Archive for Space Telescopes (https://archive.stsci.edu/hlsp/tess-spoc)^119^. The ESPRESSO and SPIRou RV data are provided in Extended Data Table 2. The ESPRESSO observations were obtained under ESO programme ID 108.22B4.001, and the raw spectra can be obtained from the ESO Science Portal under target name TIC67512645 (https://archive.eso.org/scienceportal/home). The Magellan/FIRE spectrum (Data Tag 441942) is available from the ExoFOP-TESS archive (https://exofop.ipac.caltech.edu/tess/target.php?id=67512645). The ExTrA data (Data Tag 441923), SPECULOOS data (Data Tags 438216, 438351 and 438530), TRAPPIST data (Data Tag 438352), LCOGT data (Data Tag 438460), MuSCAT2 data (Data Tag 441940) and OSN data (Data Tag 441978) are available from the ExoFOP-TESS archive (https://exofop.ipac.caltech.edu/tess/target.php?id=67512645). The Gemini North speckle imaging data (Data Tag 441696) are available from the ExoFOP-TESS archive (https://exofop.ipac.caltech.edu/tess/target.php?id=67512645).

## Code availability

The code used to run the main Markov chain Monte Carlo analysis has been previously described in refs. ^96–98^. The SPLAT code is available from https://github.com/aburgasser/splat. The ODUSSEAS code is available from https://github.com/AlexandrosAntoniadis/ODUSSEAS. The PROSE code is available from https://github.com/lgrcia/prose. AstroImageJ is described in ref. ^65^ and is available from https://www.astro.louisville.edu/software/astroimagej/. The BANZAI code is described in ref. ^67^ and is available from https://github.com/LCOGT/banzai. The MuSCAT2 data reduction pipeline is described in ref. ^69^. The FIRE bright source data reduction pipeline is described in ref. ^70^. The ESPRESSO data-reduction pipeline is available from https://www.eso.org/sci/software/pipelines/espresso/espresso-pipe-recipes.html. The APERO pipeline is described in ref. ^79^ and is available from https://github.com/njcuk9999/apero-drs.
