# Supplementary material for: A transiting giant planet in orbit around a 0.2-solar-mass host star
Source: Nat Astron. 2025 Jun 4;9(7):1031–44. doi: 10.1038/s41550-025-02552-4 (PMC12274134; doi:10.1038/s41550-025-02552-4)
Supplement: Supplementary file 1 — Supplementary Figs. 1–3 and Tables 1 and 2. [file 41550_2025_2552_MOESM1_ESM.pdf]

nature astronomy

Supplementary information

https://doi.org/10.1038/s41550-025-02552-4

A transiting giant planet in orbit around a 0.2-solar-mass host star

In the format provided by the authors and unedited

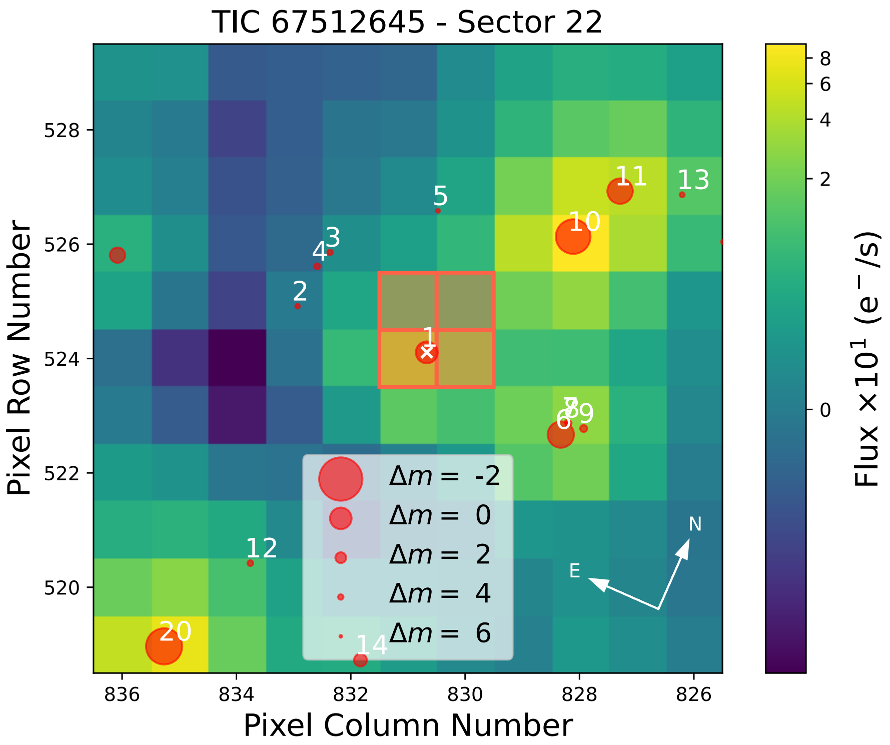

Supplementary Figure 1: A 11 × 11 pixel cutout of the TESS image aronud the location of TOI-6894 from Sector 22, plotted using TPFPLOTTER (123). The red shaded boxes highlight the aperture used by the SPOC pipeline to extract the photometric light curve, and the nearby sources from *Gaia* are labeled with the red circles. The size of each marker corresponds to the magnitude of the star in the *Gaia G* band relative to TOI-6894.

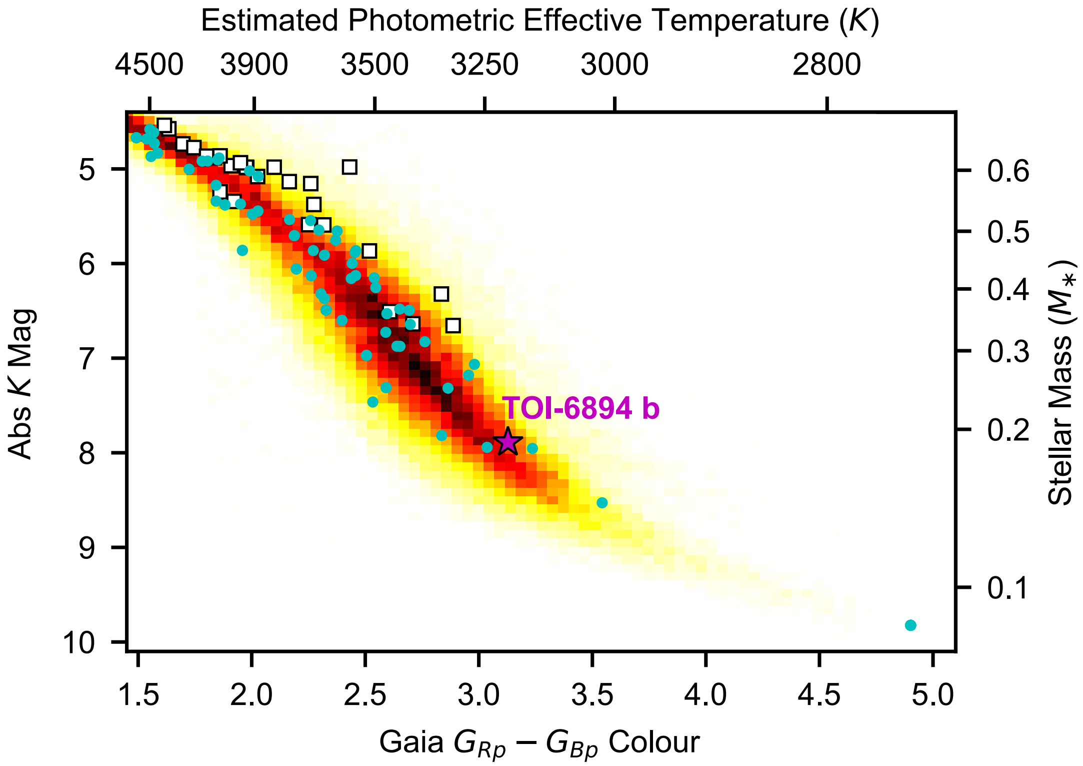

Supplementary Figure 2: Colour-magnitude diagram displaying the *Gaia* $G_{\mathrm{Bp}} - G_{\mathrm{Rp}}$ colour and absolute *2MASS K* magnitude. The 2D histogram heat map shows the distribution of the population of low-mass stars studied by (14). The individual markers show the host stars of TOI-6894 b (purple star); known transiting giant planets (black open squares; $M_{\mathrm{P}} \geq 0.1\, M_{\mathrm{J}}$); and other known transiting planets (cyan circles). The parameters of known planet host stars are taken from the NASA Exoplanet Archive (accessed 16 May 2024). The upper axis provides an approximate representation of the stellar effective temperature, using the scaling provided in (124), and the right-hand axis provides a representation of the stellar mass, computed using the scaling from (125).

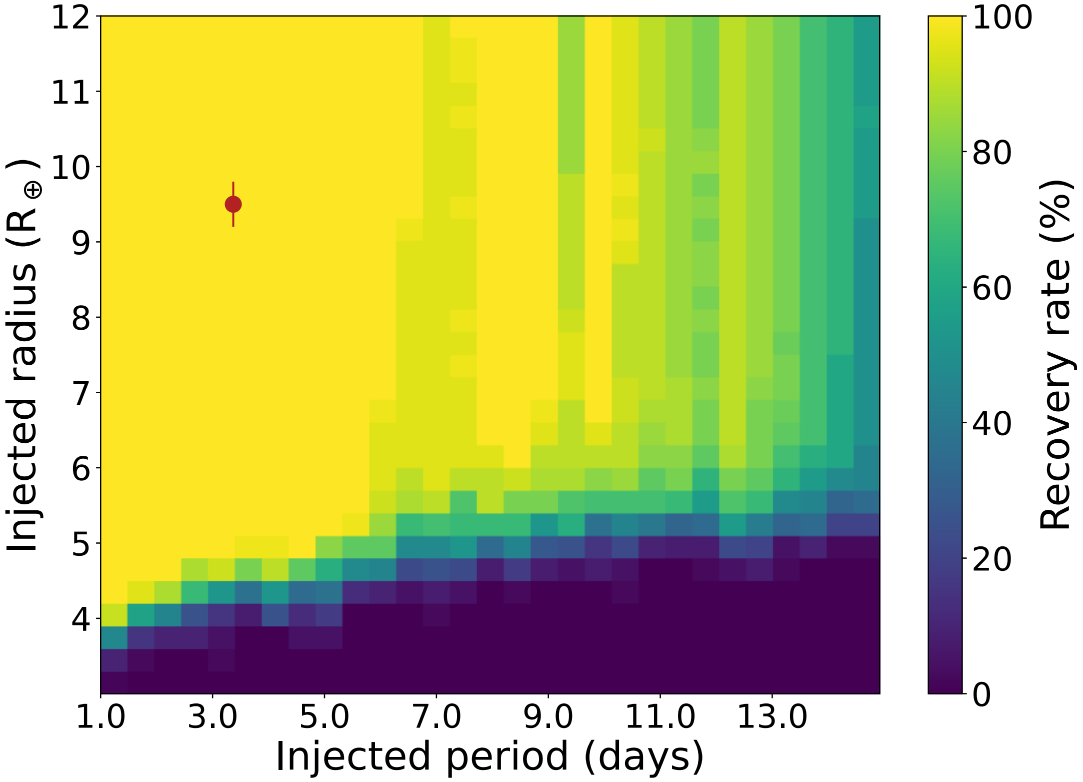

Supplementary Figure 3: Injection-and-retrieval experiment conducted to test the detectability of extra planets in the system TOI-6894 using the TESS 120 s data, corresponding to Sector 72. We explored a total of 36000 different scenarios. Each pixel shows the evaluation of about 40 scenarios, that is, 40 light curves with injected planets having different $P_{\rm planet}$, $R_{\rm planet}$, and T_0. Larger recovery rates are presented in yellow and green colors, while lower recovery rates are shown in blue and darker hues. Planets smaller than 4.0 $R_\oplus$ would be undetectable for the explored periods. The red dot refers to the planet TOI-6894 b.

Supplementary Table 1: Physical Parameters varied in joint analysis

**Parameter**	**Prior**	**Notes**
T_A	uniform	mid transit time of first observed transit
T_B	uniform	mid transit time of last observed transit
K	uniform, $K > 0$	RV semi-amplitude
$\sqrt{e}\cos\omega$	uniform, $0 \leq e < 1$	eccentricity parameter, either fixed to zero or varied
$\sqrt{e}\sin\omega$	uniform, $0 \leq e < 1$	eccentricity parameter, either fixed to zero or varied
$R_{\rm P}/R_*$	uniform	ratio of planetary to stellar radius
b^2	uniform, $b^2 \geq 0$	impact parameter squared
ζ/R_*	uniform	reciprocal of the half duration of the transit
γ_i	uniform	systemic velocity for RV instrument i
$LD_{b,j}$	Gaussian with $\sigma = 0.2$ mean based on (100; 101; 102)	Linear limb darkening coefficient for filter j
$LD_{b,j}$	Gaussian with $\sigma = 0.2$ mean based on (100; 101; 102)	Quadratic limb darkening coefficient for filter j
$d_{\rm mod}$	$2\ln(\frac{d_{\rm mod}+5}{5}) - \frac{d_{\rm mod}+5}{7650}$	distance modulus, note the *Gaia* DR3 parallax is treated as an observable to be fit
A_V	Gaussian with $\sigma = 0.25\,{\rm mag}$ mean based on MWDUST model $A_V \geq 0$	extinction
$T_{\rm eff}$	Gaussian, 2960 ± 66, $T_{\rm eff} > 0$	host star effective temperature
[Fe/H]	Gaussian, $+0.240 \pm 0.081$	host star metallicity

Supplementary Table 2: Auxiliary Parameters varied in joint analysis

**Parameter**	**Prior**	**Notes**
$\sigma_{\rm jit,i}$	$-\log(\sigma_{\rm jit,i})$, $\sigma_{\rm jit,i} > 0$	jitter for RV instrument i
$m_{0,TESS,i}$	uniform	out-of-transit magnitude for *TESS* light curve i
$d_{TESS,i}$	uniform, $0 < d_{HS,i} \leq 1$	transit dilution factor for *TESS* light curve i
$m_{0,LC,i}$	uniform	out-of-transit magnitude for follow-up light curve i
$m_{1,LC,i}$	uniform	linear trend to out-of-transit magnitude for follow-up light curve i
$m_{2,LC,i}$	uniform	quadratic trend to out-of-transit magnitude for follow-up light curve i
$\delta x_{0,LC,i}$	uniform	linear detrending coefficient for CCD Δx position of star for follow-up light curve i. Used for SPECULOOS, TRAPPIST, and OSN.
$\delta y_{0,LC,i}$	uniform	linear detrending coefficient for CCD Δy position of star for follow-up light curve i. Used for SPECULOOS, TRAPPIST, and OSN.
$fwhm_{0,LC,i}$	uniform	linear detrending coefficient for FWHM of star for follow-up light curve i. Used for SPECULOOS, TRAPPIST, and OSN.
$sky_{0,LC,i}$	uniform	linear detrending coefficient for sky background for follow-up light curve i. Used for SPECULOOS, TRAPPIST, and OSN
$\sigma_{M_*,sys}$	Gaussian with $\sigma = 5\%$	Fractional systematic uncertainty on M_*
$\sigma_{{\rm [Fe/H]},sys}$	Gaussian with $\sigma = 0.08\,{\rm dex}$	Systematic uncertainty on [Fe/H]
$\sigma_{T_{\rm eff},sys}$	Gaussian with $\sigma = 4\%$	Fractional systematic uncertainty on $T_{\rm eff}$
$\sigma_{M_{bol},sys}$	Gaussian with $\sigma = 0.021\,{\rm mag}$	Systematic uncertainty on bolometric magnitude

[123] Aller, A., Lillo-Box, J., Jones, D., Miranda, L. F. & Barceló Forteza, S. Planetary nebulae seen with TESS: Discovery of new binary central star candidates from Cycle 1. *A&A* **635**, A128 (2020).

[124] Mann, A. W., Feiden, G. A., Gaidos, E., Boyajian, T. & von Braun, K. How to constrain your m dwarf: Measuring effective temperature, bolometric luminosity, mass, and radius. *ApJ* **804**, 64 (2015).

[125] Mann, A. W. *et al.* How to Constrain Your M Dwarf. II. The Mass-Luminosity-Metallicity Relation from 0.075 to 0.70 Solar Masses. *ApJ* **871**, 63 (2019).
