# Supplementary material for: A transiting giant planet in orbit around a 0.2-solar-mass host star
Source: Nat Astron. 2025 Jun 4;9(7):1031–44. doi: 10.1038/s41550-025-02552-4 (PMC12274134; doi:10.1038/s41550-025-02552-4)
Supplement: Supplementary file 2 — Reporting Summary [file 41550_2025_2552_MOESM2_ESM.pdf]

nature portfolio

Corresponding author(s): Edward M. Bryant

Last updated by author(s): Feb 7, 2025

Reporting Summary

Nature Portfolio wishes to improve the reproducibility of the work that we publish. This form provides structure for consistency and transparency in reporting. For further information on Nature Portfolio policies, see our Editorial Policies and the Editorial Policy Checklist.

Statistics

For all statistical analyses, confirm that the following items are present in the figure legend, table legend, main text, or Methods section.

n/a	Confirmed	
☒	☐	The exact sample size (*n*) for each experimental group/condition, given as a discrete number and unit of measurement
☒	☐	A statement on whether measurements were taken from distinct samples or whether the same sample was measured repeatedly
☒	☐	The statistical test(s) used AND whether they are one- or two-sided<br>*Only common tests should be described solely by name; describe more complex techniques in the Methods section.*
☒	☐	A description of all covariates tested
☒	☐	A description of any assumptions or corrections, such as tests of normality and adjustment for multiple comparisons
☒	☐	A full description of the statistical parameters including central tendency (e.g. means) or other basic estimates (e.g. regression coefficient) AND variation (e.g. standard deviation) or associated estimates of uncertainty (e.g. confidence intervals)
☒	☐	For null hypothesis testing, the test statistic (e.g. *F*, *t*, *r*) with confidence intervals, effect sizes, degrees of freedom and *P* value noted<br>*Give P values as exact values whenever suitable.*
☐	☒	For Bayesian analysis, information on the choice of priors and Markov chain Monte Carlo settings
☒	☐	For hierarchical and complex designs, identification of the appropriate level for tests and full reporting of outcomes
☒	☐	Estimates of effect sizes (e.g. Cohen's *d*, Pearson's *r*), indicating how they were calculated

Our web collection on statistics for biologists contains articles on many of the points above.

Software and code

Policy information about availability of computer code

Data collection	The PROSE code is available https://github.com/lgrcia/prose; AstroImageJ is described in (73) and available from https://www.astro.louisville.edu/software/astroimagej/; the BANZAI code is described in (75) and available from https://github.com/LCOGT/banzai; the MuSCAT2 data reduction pipeline is described in (77); the FIRE bright source data reduction pipeline is described in (78); the ESPRESSO DRS pipeline is available from https://www.eso.org/sci/software/pipelines/espresso/espresso-pipe-recipes.html; the APERO pipeline is described in (84) and is available from https://github.com/njcuk9999/apero-drs.
Data analysis	The code used to run the main MCMC analysis has been previously described in (102; 103; 104). The SPLAT code is available from https://github.com/aburgasser/splat and the ODUSSEAS code is available from https://github.com/AlexandrosAntoniadis/ODUSSEAS.

For manuscripts utilizing custom algorithms or software that are central to the research but not yet described in published literature, software must be made available to editors and reviewers. We strongly encourage code deposition in a community repository (e.g. GitHub). See the Nature Portfolio guidelines for submitting code & software for further information.

Data

Policy information about availability of data

All manuscripts must include a data availability statement. This statement should provide the following information, where applicable:

- Accession codes, unique identifiers, or web links for publicly available datasets
- A description of any restrictions on data availability
- For clinical datasets or third party data, please ensure that the statement adheres to our policy

The TESS photometry is publicly available from the Mikulski Archive for Space Telescopes (MAST; https://archive.stsci.edu/missions-and-data/tess). The ESPRESSO and SPIRou RV data is provided in Table 2 in the Supplementary Information within this paper. The ESPRESSO data were obtained under ESO programme ID 108.22B4.001. The Magellan/FIRE spectrum (Data Tag 441942) is available via the ExoFoP-TESS archive (https://exofop.ipac.caltech.edu/tess/target.php?id=67512645). The ExTrA data (Data Tag 441923), SPECULOOS data (Data Tags 438216, 438351, and 438530), TRAPPIST data (Data Tag 438352), LCOGT data (Data Tag 438460), MuSCAT2 data (Data Tag 441940), and OSN data (Data Tag 438460) are available via the ExoFoP-TESS archive (https://exofop.ipac.caltech.edu/tess/target.php?id=67512645). The Gemini North speckle imaging data (Data Tag 441696) is available via the ExoFoP-TESS archive (https://exofop.ipac.caltech.edu/tess/target.php?id=67512645).

Research involving human participants, their data, or biological material

Policy information about studies with human participants or human data. See also policy information about sex, gender (identity/presentation), and sexual orientation and race, ethnicity and racism.

Reporting on sex and gender	There were no human participants involved in the study.
Reporting on race, ethnicity, or other socially relevant groupings	There were no human participants involved in the study.
Population characteristics	There were no human participants involved in the study.
Recruitment	There were no human participants involved in the study.
Ethics oversight	There were no human participants involved in the study.

Note that full information on the approval of the study protocol must also be provided in the manuscript.

Field-specific reporting

Please select the one below that is the best fit for your research. If you are not sure, read the appropriate sections before making your selection.

☒ Life sciences ☐ Behavioural & social sciences ☐ Ecological, evolutionary & environmental sciences

For a reference copy of the document with all sections, see nature.com/documents/nr-reporting-summary-flat.pdf

Life sciences study design

All studies must disclose on these points even when the disclosure is negative.

Sample size	The MCMC sampling sizes were determined by ensuring the chains had converged before the sample was finalised
Data exclusions	Some ground-based photometric points were excluded as the images were heavily impacted by clouds. This is discussed in the text.
Replication	As this is a discovery paper for a new exoplanet, reproducibility in the paper is not appropriate. However we ensure all data is publicly available and our methods are discussed and the codes used are appropriately referenced and cited so other researchers will be able to reproduce our analyses and results
Randomization	This is not relevant to this study as it is reporting the discovery of a single planet.
Blinding	Blinding was not relevant to this study as it is reporting the discovery of a single planet.

Reporting for specific materials, systems and methods

We require information from authors about some types of materials, experimental systems and methods used in many studies. Here, indicate whether each material, system or method listed is relevant to your study. If you are not sure if a list item applies to your research, read the appropriate section before selecting a response.

Materials & experimental systems

n/a	Involved in the study
☒	☐ Antibodies
☒	☐ Eukaryotic cell lines
☒	☐ Palaeontology and archaeology
☒	☐ Animals and other organisms
☒	☐ Clinical data
☒	☐ Dual use research of concern
☒	☐ Plants

Methods

n/a	Involved in the study
☒	☐ ChIP-seq
☒	☐ Flow cytometry
☒	☐ MRI-based neuroimaging

Plants

Seed stocks	Plants were not involved in this study
Novel plant genotypes	Plants were not involved in this study
Authentication	Plants were not involved in this study
